# Infective Complications of Endobronchial Ultrasound-Transbronchial Needle Aspiration (EBUS-TBNA) and Clinical Biomarkers: A Concise Review

**DOI:** 10.3390/diagnostics15020145

**Published:** 2025-01-09

**Authors:** Pinelopi Bosgana, Dimitrios Ampazis, Vasileios Vlachakos, Argyrios Tzouvelekis, Fotios Sampsonas

**Affiliations:** 1 Department of Pathology, General Hospital of Patras, 26504 Patras, Greece; bosgana.p@gmail.com; 2 Respiratory Department Cavan & Monoghan Hospital, HSE/RCSI Hospital Group, H12Y7W1 Cavan, Ireland; dim_ampazis@yahoo.gr; 3 Bioclinic General Hospital of Athens, Henry Dunant Hospital Center, 11526 Athens, Greece; vasvlahakos@gmail.com; 4 Department of Respiratory Medicine, Medical School, University of Patras, 26504 Patras, Greece

**Keywords:** EBUS-TBNA, infective complications, biomarkers, interleukin-6, procalcitonin

## Abstract

EBUS-TBNA is the most common interventional pulmonology procedure performed globally and remains the cornerstone of the diagnosis and staging not only of lung cancer but also for other neoplastic, inflammatory, and infective pathologies of the mediastinum. Infective complications of EBUS-TBNA are underreported in the literature, but the constantly rising incidence of lung cancer is leading to an increasing number of EBUS-TBNA procedures and, therefore, to a significant number of infective complications, even 4 weeks following the procedure. In this review we attempt to summarize the risk factors related to these infective complications, along with useful biomarkers that can be used to identify patients that might develop infective complications, to facilitate the prediction or even prompt treatment of these.

## 1. Introduction

Endobronchial ultrasound (EBUS)-guided transbronchial needle aspiration biopsy (TBNA/B) with or without bronchoscopic, though the EBUS bronchoscope, esophageal ultrasound Fine Needle Biopsy (EUS-B-FNB) have been cornerstones in the diagnosis and staging of lung cancer and other mediastinal (neoplastic, infective, and inflammatory lesions of the mediastinum) over the last two decades [1,2]. EBUS-TBNA and EUS-B-FNB technics are preferable over the surgical mediastinal sampling since they are minimally invasive procedures that can be performed without general anesthesia, offering prompt, relatively cheap diagnosis with low reported complication rates [1,3,4].

Lung cancer remains a malignancy with high mortality despite the recent treatment advances [5,6,7,8,9,10]. Early diagnosis and accurate clinical and pathological staging are pivotal for prognostic assessment and important to define treatment plans for lung cancer patients [11,12,13,14,15,16,17]. In early-stage lung cancer, treatment includes surgical resection, and in more advanced disease, options are systemic therapy and radiotherapy [18,19,20]. The localization and size of the tumor, the absence of distant metastasis, and the level of thoracic lymph node engagement determine the possibility of surgical resection and remain the best predictors of mortality [21]. The sampling of mediastinal lymph nodes is therefore crucial for both diagnosis and staging lung cancer but also for the diagnosis of other mediastinal diseases (sarcoidosis, tuberculosis, lymphoma, extra-thoracic metastatic malignancies, etc.) [18,22] (Table 1). EBUS-TBNA is a safe procedure but, rarely, can cause infective complications. In this review, we present the infective complications of the procedure and the useful clinical biomarkers for these complications.

## 2. EBUS-TBNA and Targeted Therapy in Lung Cancer

The American College of Chest Physicians guidelines recommend EBUS-TBNA as the procedure of choice for the diagnoses and staging of lung cancer [11]. Patients with centrally located tumors larger than 3 cm, or PET negative primary tumors in the cases of those who undergo surgical resection, should be staged preoperatively using EBUS TBNA as recommended by the combined guidelines of the European Respiratory Society, the European Society of Gastrointestinal Endoscopy, and the European Society of Thoracic Surgeons [24]. Histological and cytological EBUS-TBNA samples can be used for molecular testing and Next-Generation Sequencing technique [25]. Newer therapeutic agents targeting driver gene mutations for Non-Small-Cell Lung cancer (mainly adenocarcinomas) are the treatment of choice over the last two decades [26], such as osimerdinib and alectinib (for EGFR mutations and ALK rearrangements) [27,28,29]. At the same time, patients with significant PDL-1 expression (>50%) can be treated with immune checkpoint PD-1 inhibitors, like pembrolizumab, as a first-line treatment [19,30,31,32]. These therapies can increase the survival rate in both patients with advanced and limited disease [33,34]. Thus, it is important to obtain adequate tissue samples, in a timely manner, for driver gene mutation and PDL-1 expression [35].

In this context, thousands of EBUS-TBNA procedures are performed annually, making EBUS-TBNA the most common interventional procedure in respiratory medicine. The scope of this review is to summarize and present the inflammatory—mainly infective—complications related to this procedure, since they can be grossly underestimated and overlooked.

## 3. Infective Complications of EBUS-TBNA

There are a few studies, usually case reports and case series [36], reporting mediastinitis and infective pericarditis following EBUS-TBNAs but systematic retrospective and prospective studies systematically reporting the infective complications are scarce. One of the biggest studies dealing with the inflammatory reaction and infectious complications of EBUS-TBNA was the retrospective, single-center study by Chen M. et al. that investigated 512 patients who underwent bronchoscopy and/or EBUS [37]. Three study groups were evaluated—one with patients having bronchoscopic inspection only, one with conventional bronchoscopy and conventional sampling, and one with bronchoscopy followed by sampling of the mediastinum with EBUS-TBNA. Inflammatory biomarkers were assessed from the peripheral blood, including white blood cells, neutrophils, and interleukin 6 (IL-6) before and after the procedure. For the patients that developed post-procedural fever (defined as temperature more than 38.5 °C), blood cultures were also obtained. The temperature activity was also monitored, recording onset time, average duration, and peak values [37]. The inspection-only bronchoscopy group had lower feverish activity compared to the other groups. The differences, however, between the group of conventional bronchoscopy sampling and the group with EBUS-TBNA sampling were not statistically significant. In all three groups, the inflammatory markers (white blood cells, neutrophils, and IL-6) increased post procedure. The inflammatory markers of the inspection-only group, however, despite being raised, remained within normal limits. In both groups that had sampling, the inflammatory markers were higher after the procedure, with the EBUS-TBNA group having more increased values [37]. In relation to the patients that developed fever, for whom blood cultures were obtained, 15 samples came back as positive. Of those, 13 (8 from the conventional bronchoscopy group and 5 from the EBUS-TBNA group) had no clinical findings other than fever. Two patients, however, of each group experienced a productive cough along with the fever, with the cultures identifying *Staphylococcus aureus* and *Streptococcus pneumoniae* as the causative pathogens. Parameters such as lesion size, sampling effort, and pathological findings did not correlate with bacteremia. The only common factor identified in the two patients was diabetes mellitus. The study could not identify other statistically significant differences between the groups of conventional bronchoscopy sampling and EBUS-TBNA. According to this study, the authors suggest that postoperative fever after EBUS-TBNA is a common complication that is not related to bacterial infection. This is due to a systemic inflammatory reaction to the procedure that does not need antibiotic treatment [37].

Similarly, in a retrospective single-center study, Moon K. et al. evaluated data from the medical records for 6336 patients who underwent EBUS-TBNA over a period of 10 years [38]. The primary parameter evaluated was the development of post-procedure fever that occurred within 24 h, defined as a temperature above 37.8 °C. Out of 6336 patients, 665 (10.5%) experienced fever, with the mean peak temperature being at 38.3 °C. In the fever group, more EBUS-TBNA samples had been obtained (mean: 2.14 vs. 2.03 between the fever and non-fever groups, *p* = 0.004), especially in those patients from whom more than four samples were obtained (5.7% vs. 3.8% respectively, *p* = 0.021) [38]. Patients in the fever group had more frequent diagnosis of tuberculosis compared to the non-fever group (7.5% vs. 2.9% respectively, *p* < 0.001). The group with fever had also undergone more interventions in addition to EBUS-TBNA, such as bronchial washing, endobronchial biopsy, core needle biopsy, and transbronchial biopsy [38]. Beyond these, additional risk factors associated with post-procedure fever were older age, lower pre-procedural Hb, and higher pre-procedural CRP. The authors’ explanation for the fever development was transient bacteriemia due to contamination of the sampling needle by oropharyngeal bacteria [38]. This study suggests that fever after EBUS-TBNA is the cause of transient bacteriemia by oropharyngeal bacteria, like the previous studies of Huang C.T and Haas A.R have shown [39,40].

Kim S.Y. et al. also reviewed 684 patients, over a period of 2 years, that underwent EBUS-TBNA [41]. Their objective was to identify the post-procedural occurrence of fever within the first 24 h. In total, 552 patients met the inclusion criteria for the final analysis. The incidence of fever was 20%; the median time of fever onset was 7 h; and the median duration was 7 h. Fever exceeded 24 h in duration in six cases (1.1%). Infectious complications were identified in three cases (0.54%) [41]. The study did not reveal any significant correlation between fever development and risk factors such as higher age, abnormal endobronchial findings, sampling with BAL, bronchial washing or biopsy, number of lymph nodes sampled, or necrotic features of the lymph nodes. The only common characteristic of the three cases who developed infectious complication was diabetes [41].

In an analogous multi-center study, Asano F. et al. analyzed data from 520 institutions regarding EBUS-TBNA-related complications [42]. The data collection was facilitated via a questionnaire. The study focused on EBUS-TBNA-only complications excluding multi-procedural approaches. Out of the 520 centers involved, 455 provided responses and 210 of them had results relevant to EBUS-TBNA, reflecting data from 7345 cases [42]. EBUS-TBNA complications were identified in 90 cases. The most common complication reported was hemorrhage, in 50 cases (0.68%). Infective complications (mediastinitis, pneumonia, pericarditis, sepsis, and cyst infection) developed in 14 cases (0.19%) [42]. Other complications involved respiratory failure (five cases, 0.07%), pneumothorax (two cases, 0.03%), lidocaine toxicity (four cases, 0.05%), asthmatic attack (one case, 0.01%), cardiac arrhythmia (three cases, 0.04%), hypotension (one case, 0.03%), fever (four cases, 0.05%), cerebral infarction (two cases, 0.03%), aggravation of airway obstruction (two cases, 0.03%), tumor rupture (one case, 0.01%), and hyperventilation syndrome (one case, 0.01%) [42]. Of the abovementioned complications, 57 cases had no further adverse events relevant to them. Life-threatening events were observed in four cases, namely mediastinitis (two cases), tumor rupture (one case), and airway obstruction (one case). Death was the outcome in one case (1.3% of complications) due to cerebral infarction, providing a global mortality rate of 0.01% [42]. Operator experience seemed to be related to the rate of complications; slightly higher rates were observed with less experienced operators. EBUS scope damage was higher in this study compared to other similar studies [42].

Kang N. et al. studied 6826 patients [43], reflecting a period of 10 years and ranging in follow-up period from 2 months for each case following EBUS-TBNA. The objective of the study was to identify infectious complications and the relevant risk factors. The infectious complication incidence was 0.5% and the risk was significantly increased in cases with necrotic features of the target lesion and when EBUS-TBNA was combined with EBUS-B-FNB [43]. The median number of days of infectious-related clinical findings, warranting antibiotic initiation, was seven. This was slightly longer compared to post-procedural pneumonia, indicating the relative delay in recognizing infectious complications of the mediastinum [43].

A rather small but prospective study by Steinfort D.P et al. included 43 patients with the objective of identifying post-EBUS-TBNA infectious complications [44]. Assessment included clinical evaluation and post-procedural blood sampling 60 min after EBUS-TBNA. Samples of the EBUS-TBNA needle were also obtained. Of the 43 patients, 3 (7%) developed bacteriemia but none of them experienced any significant complications [44]. The pathogens identified were related to the oropharyngeal flora. There was no significant correlation between bacteriemia and the size of the sampled lesion or the underlying pathology. The bacteriemia rate was comparable to that induced by conventional bronchoscopy. The cultures of TBNA needle washings were negative in all three cases of bacteriemia [44]. Nevertheless, the sample size was too low to derive significant outcomes.

A single-centered prospective study by Magnini et al. evaluated post-bronchoscopy/endonosonography (EBUS and EUS-B) complications within a period of 30 days [45]. The study included 697 patients that underwent procedures in a 15-month period. The primary outcome focused on major and severe complications such as respiratory failure, infection, and bleeding. Secondary outcomes of the study included parameters such as unplanned hospital encounters, 30-day mortality, adverse events by procedure type, and factors associated with adverse events. Severe complications were identified in a significant 2.4% (17) of cases. Some of the severe complications (8.47%) occurred late in the post-procedural follow-up period, with a median of 14 days [45]. Infective complications only occurred in patients with malignancy. The infectious complications that led to unplanned hospital encounters accounted for 2.5% of the cases [45]. Interestingly, these infectious complications had a significant negative impact, both clinical and financial, since they led to prolonged (>2 weeks) use of antibiotics and delayed oncological treatment. Lesions with low-density areas had increased the likelihood of developing infectious complications [45]. The 30-day mortality rate related to EBUS-TBNA reached an astonishing 0.29% [27].

Souma T. et al. reviewed 1045 patients that underwent EBUS-TBNA within a 4-year period. The aim of this study was to identify infectious complications after sampling peripheral lesions via a guide sheath [46]. Out of 1045 cases, 47 (4.5%) developed relevant complications such as pneumonia (24), intratumoral infection (14), lung abscess (3), pleuritis (3), and empyema (3). The main risk factors identified were cavitation of the lesion, low-density areas in the lesion, and bronchial stenosis [46]. The authors suggested that the above risk factors were likely related to the inflammation-prone status of the lesions and, thus, an increased likelihood of post-sampling infection. The use of prophylactic antibiotics, just before or after the procedure, in 102 patients could not provide reliable results regarding the efficacy of preventing post-procedural infectious complications [46].

A very interesting study by Minami D et al. evaluated, retrospectively, 80 cases that underwent EBUS-TBNA [47]. The study split the population in two groups comprising 60 cases that had EBUS-TBNA via endobronchial intubation and 20 cases that had EBUS-TBNA without intubation. The study focused on EBUS-TBNA needle wash cultures [47]. The intubated group had positive cultures in only 2 cases (3.3%) while the non-intubated group developed positive cultures in all 20 cases (100%). An interesting finding, however, is that among the intubated cases, six (10%) developed fever, while in the non-intubated group, only two cases (10%) developed fever [47]. The above finding suggests that, despite contamination of the EBUS-TBNA needle being less likely with the use of intubation, fever development was equal in both groups [47], suggesting an inflammatory, non-infective etiology of the feverish reaction. In support of the above, in a single-center, assessor-blinded, parallel-group randomized controlled trial, where participants were allocated to either sterilize their oral cavity with oral chlorhexidine or with no chlorhexidine, no statistically significant differences were found in the incidence of fever, infective complication rates, or positive EBUS bronchoscope rinse cultures [48].

One of the few prospective studies was by Mitja et al. [49], in which 245 patients with risk factors (immunosuppression, cavitary, or necrotic lung lesions; multiple TBNA biopsies; or chronic bacterial colonization) were compared against 125 patients with no risk factors (control group). The overall infectious complication rates were 4.05% (15 patients), of which 14 were patients with risk factors and 1 was from the control group. Subgroup analysis showed that patients with risk factors and necrosis in the biopsied lesions were more prone to the development of complications (*p* = 0.018) [49].

In summary, postoperative fever is a common complication that clinicians have to manage, but in all reported cases, this was temporary and did not cause major problems. The significantly associated factors were bronchoscopic washing, older age, low hemoglobin levels, high CRP levels, and tuberculosis. Patients with diabetes and prolonged fever after 24 h developed pneumonia. Furthermore, very rare life-threating complications include mediastinitis, pericarditis, and sepsis. All of these cases were treated with antibiotics and had good prognosis. Another very rare complication is cystic infection. Patients with malignancy, who had infection complications following EBUS-TBNA, had delayed treatment of neoplastic disease because of the prolonged treatment of infection. In all of these cases, the infectious complications resolved with antibiotics without any more severe complication. Thus, EBUS-TBNA is a safe procedure but, rarely, can cause mild to severe complications. All authors suggest that prophylactic antibiotics have no use.

Some differences in the results of previous studies may be due to patient populations or sample size. A systemic metanalysis of the discussed studies was not performed because of the diversity and the heterogeneity of the population. Table 2 summarizes the complications reported in each study, with information about the inflammation biomarkers in patients’ blood samples.

## 4. Laboratory Biomarkers That Can Be Used to Predict EBUS-TBNA Related Infective Complications

Sepsis is a severe complication of infection and a leading cause of death in hospitalized patients and is associated with a high mortality rate [50]. In 2017, 49 million patients had sepsis worldwide and 11 million of them died [50,51]. The first clinical signs are non-specific, such as fever and leukocytosis, and, in the progression of the disease (severe sepsis), arterial hypotension. Early therapy in the first hours of sepsis can decrease mortality [52]. Thus, it is vital to have useful biomarkers with sensitivity, specificity, and low cost for early diagnosis of the condition, especially in procedural-related septic reactions [52].

**Figure 1:**
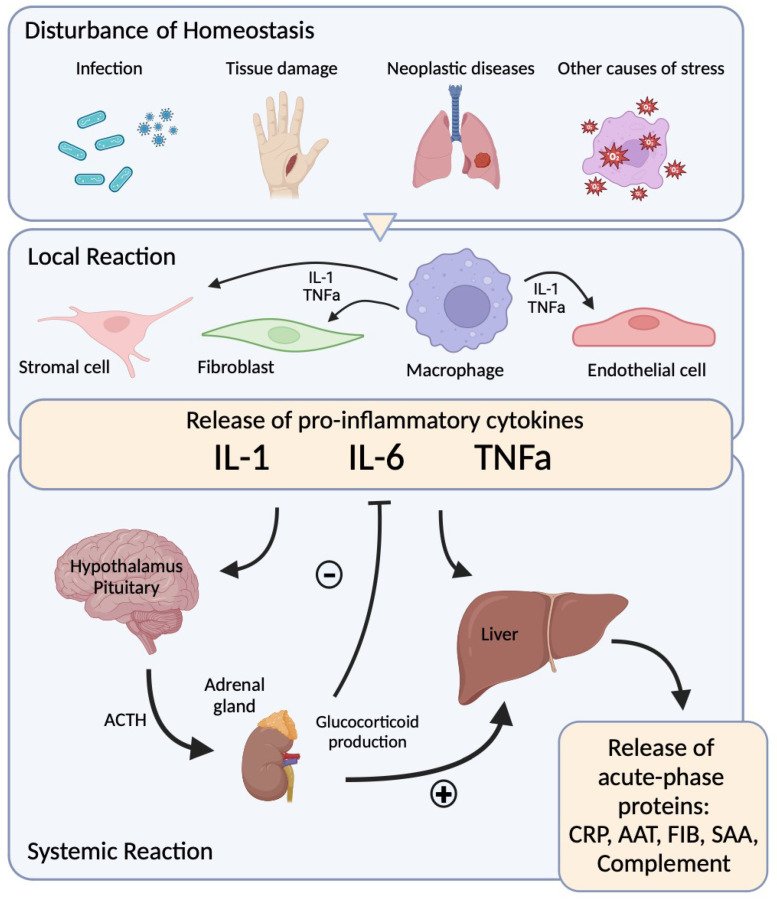
Pro-inflammatory cytokine release related to acute infection, tissue damage, neoplastic disease, and stress.

The acute response is the answer of the human body to tissue injury, cancer, immunological disorders, and infection to maintain homeostasis [53]. The inflammatory reaction consists of humoral, cellular, and molecular pathways [54,55]. In response to infection, tissue injury, or neoplasia, several cell-activation molecules and proteins are produced (Figure 1) [56,57]. Bacteria and viruses stimulate monocytes and macrophages, which synthesize cytokines IL-1, IL-6, and TNFa [58,59]. These react with hepatic receptors in hepatocytes and produce acute-phase proteins (Figure 1). IL-6 is the regulator of the acute-phase synthesis in the liver [56,60]. The cytokines IL-I and TNFa also stimulate endothelial cells and fibroblasts as a local reaction to inflammation. IL-6, IL-1, and TNFa control the release of ACTH from the pituitary cells of the brain. This results in the secretion of glucocorticoids by adrenocortical cells. The glucocorticoids stimulate the synthesis of acute-phase proteins and inhibit the production of cytokines [61].

A biomarker is any molecule that can be measured in the body, which can predict the incidence of a disease [62,63]. Biomarkers can be used to monitor a patient’s response to infection by mediating the response to treatment, and they allow earlier identification of patients with severe infections and help to choose rapidly the appropriate treatment [64,65]. A diagnostic biomarker for infection should be very low or absent when inflammation is absent and high in the presence of infection [66]. Furthermore, the ideal biomarker should provide results sooner than a blood culture and should have high specificity or sensitivity [67,68,69]. Therefore, we report useful biomarkers that can be potentially used in infections related to EBUS-TBNA procedures (Table 3).

## 5. Interleukin 6

Interleukin 6 (IL-6) is a cytokine involved in different biological events [71] associated with inflammatory processes, autoimmune diseases, and lymphoproliferative disorders [72,73,74]. IL-6 is a small glycoprotein with 184 amino acids, which is composed of four helixes [75]. The biological functions of IL-6 are achieved via two pathways—the classic signaling pathway and the trans-signaling pathway. In the classic pathway, IL-6 binds to the IL-6 receptor on hepatocytes and leukocytes. This complex triggers the dimerization of gp130 and the signaling in the cell. In the trans-signaling pathway, IL-6 reacts with IL-6R (sIL-6R) and forms the IL-6_ sIL-6R complex. This binds to gp130 on cells and plays a role in intracellular signaling [76,77]. The Sil-6R complex is normally present in blood at 50 ng/mL [78]. The soluble receptors have higher levels than IL-6 in blood (1–5 pg/mL) [78] and mediate IL-6 inflammation [79].

RALI-Dx is a “rapid acute lung injury diagnostic assay” that can be used in order to quantify IL-6 (along with IL-8 and IL-10), soluble tumor necrosis factor receptor 1 (sTNFR1), and soluble triggering receptor expressed on myeloid cells 1 (sTREM1). In the study by Husain S et al., these immune activation markers were correlated with the body’s response to respiratory tract infections [80]. This study identified IL-6 (as part of RALI-Dx) as a critical biomarker of lung injury evaluation [81,82,83]. Also, the results of previous studies confirm that these biomarkers of lung injury and ARDS are useful for evaluating responses to pulmonary infections [80,84]. In the study by Chen M. et al. [22], Il-6 levels were increased within 2 h in patients that had had tissue biopsies (either conventional or EBUS-TBNA), highlighting the association between IL-6 snf acute bacteremia, tissue damage, or both.

## 6. Procalcitonin

Procalcitonin (PCT) is the precursor peptide of the calcitonin hormone [85]. It is produced by the parafollicular cells (C cells) of the thyroid gland and by pulmonary and intestinal neuroendocrine cells [86,87]. Normal blood levels of procalcitonin are low (<0.1 ng/mL) [88]. The blood levels of procalcitonin rise in cases of bacterial infection within 4–12 h, within the range of 22–35 h [89]. The variance of procalcitonin levels between patients with microbial infections and healthy people indicate that PCT is a useful biomarker for bacterial infection and can guide antibiotic therapy [90].

Higher levels of procalcitonin are found in cases of bacterial infections in addition to viral infections and other inflammatory diseases [91,92]. This makes procalcitonin a marker with high specificity for choosing antibiotic therapy [90,93,94]. PCT could be a promising biomarker of iatrogenic inflammation related to EBUS-TBNA. In 1998, Brunkhorst and colleges reported a case of iatrogenic sepsis in which the kinetics of procalcitonin were described after injection of gram-negative bacteria [95]. In the absence of hypercalcitoninemia, elevated levels of PCT are specific markers of the severity of bacterial and fungal infections [96,97]. Dandona et al. described a rapid PCT increase within 2–4 h of injection 4 mg/kg of endotoxin [98]. In the same study, IL-6 levels increased at 3 h and PCT levels peaked at 6 h [98].

## 7. C-Reactive Protein

C-reactive protein (CRP) is a protein that is produced by the liver in response to cytocines (particularly IL-6) [99]. CRP is a biomarker of inflammation [100,101]. The secretion of CRP starts 4–6 h and peaks at 36–50 h [99]. The role of CRP in the diagnosis of inflammation, sepsis, and bacteremia has been criticized because of the delay in response to clinical stimulus and poor specificity, because it also rises in many immunologically mediated inflammatory diseases [102,103]. However, CRP levels are useful for assessing the response to antibiotics [104,105].

## 8. Conclusions

EBUS-TBNA is the most common interventional pulmonology procedure globally. Severe infective complications are not frequently reported but, due to the vast number of procedures performed globally, their number is significant, probably underreported, and the consequences can be detrimental. Hypoechoic and necrotic malignant lesions, diabetes mellitus, tuberculosis, immunosuppression of the patient, the number of biopsies obtained, EUS-B FNBs, and pre-procedural increases in CRP seem to be related to increased infective complications rates. Mouth rinses and rigid intubation prior to the procedure have not exhibited any benefit in reducing the incidence of post-procedural fever and infection rates. Biomarkers like IL-6 and possibly PCT can identify those patients at risk of severe post-procedural infective complications and can guide preventive measurements like antibiotic administration during or immediately after the procedure.

## Figures and Tables

**Table 1:** Indications for EBUS-TBNA [2,23].

Diagnostic	Lung cancer
	Granulomatous lymphadenopathy (sarcoidosis and tuberculosis)
	Lymphoma
	Extra-thoracic malignancies with mediastinal lymph node involvement
	Aspiration of thyroid lesions
	Sampling of left adrenal gland lesions
Therapeutic	Drainage of mediastinal cystic lesion [23]
	Transbronchial needle injection of chemotherapeutic agents
Special situations	Intravascular (IVNA) and Trans-vascular sampling of mediastinal lesions (TVNA)
	Mediastinal pleural nodules/thickening
	Pre-para vertebral soft tissue lesions
	Pericardial effusion
	Intra-cardiac mass
	Pulmonary embolism

**Table 2:** EBUS-TBNA inflammatory complications.

Study Authors	Study Details	Objective of Study	Main Findings	EBUS-Induced Inflammation Clinically Significant	EBUS-Induced Inflammation Correlated with Biomarkers	EBUS-Induced Inflammation Correlated with Infective Element
Chen M. et al. [37]	Retrospective Single-centered 512 patients Three study groups: - Bronchoscopy: Inspection only - Bronchoscopy: Conventional sampling - Bronchoscopy + EBUS Inflammatory biomarkers utilized: pre- and post-procedural WBC neutrophils IL-6 Post-procedural fever (>38.5 °C) Blood cultures (when fever observed)	Assessment of procedural-related inflammatory reactions and identification of infective component	All three groups had raised inflammatory biomarkers post procedure Less fever episodes with inspection-only bronchoscopy Conventional bronchoscopy sampling and EBUS sampling induced fever but with no statistically significant difference EBUS sampling had higher levels of inflammatory markers but not statistically significant Post-procedural cultures obtained in fever cases but positivity was poorly correlated to clinical significance	NO	YES Increased: WBC, Neutrophils, IL-6 Not statistically significant	NO
Moon K. et al. [38]	Retrospective Single-centered 6336 patients EBUS-TBNA cases +/− other interventions Post-procedural fever (>37.8 °C) within 24 h Inflammatory biomarkers utilized: WBC neutrophils CRP	Post-procedural fever assessed within 24 h and correlation with EBUS-TBNA sampling was evaluated	665 cases developed fever (10.5%) Mean peak temperature: 38.3 °C Fever was more frequent when samples obtained were >4/caseTB diagnosis was more frequent in the fever group 72 cases had received prophylactic antibiotics in both the fever and non-fever groups Prophylactic antibiotics had been used more frequently (5.7%) for the fever group vs. the non-fever group (0.6%), *p* < 0.001 EBUS-TBNA + other interventions induced fever more frequently (bronchial washing, endobronchial biopsies, bronchial washing, core needle biopsy, transbronchial biopsy)	YES - Fever identified - Fever group had higher 90 d mortality	YES Increased CRP, WBC, Neutrophils were significantly higher, in the fever group, pre-procedurally	YES - Transient bacteraemia - Oropharyngeal flora
Kim S.Y. et al. [39]	Retrospective Single-centered 684 patients Post-procedural fever Inflammatory biomarker utilized: WBC neutrophils	Incidence of post-procedural fever within 24 h	552 patients met criteria Incidence of fever: 110 cases, 20% Median peak temperature: 38.3 °C Median time of fever presentation: 7 h Median time of fever duration: 7 h	YES - 110 patients with fever - 34 patients no symptoms - 43 patients received antibiotics - Fever resolved within 24 h for 94.5.% of cases - 6 patients with prolonged fever >24 h, 2 developed pneumonia - The only common characteristic in 3 out of the 6 cases was diabetes - Frank infective complication in 3 cases, 0.54%	YES - Increased WBC and neutrophils at the fever group	NO - 56 cases (50.9%) were investigated with blood cultures - 1 case revealed Streptococcus hominis but was attributed to contamination - No frank bacteriaemia confirmed
Asano F. et al. [40]	Retrospective multi-centered 7345 patients EBUS-TBNA related complications reported in 90 cases Post-procedural fever assessed Inflammatory biomarkers utilized: none	Identification of most common EBUS-TBNA related complications	Infectious-related complications were the second most common (14 cases, 0.19%) after hemorrhage (50 cases, 0.68%) Prophylactic antibiotics were used in only 3 out of the 14 cases	YES - Mediastinitis - Pneumonia - Pericarditis - Sepsis - Cyst infection - Two infective complications were life-threatening - Therapeutic antibiotics were required	NO - Not reported/ described in the study	YES - Naso/oropharyngeal commensals - Poor disinfection of scope
Kang N. et al. [41]	Retrospective Single-centered 6826 patients Nested case-control study Two-month follow-up after procedure Inflammatory biomarkers utilized: none	Identification of infectious complications and of the relevant risk factors	Infectious incidence: 33 cases, 0.48% Infectious complications primarily occurred after sampling target lesions with necrotic features Infectious complications comprised of pneumonia and mediastinal infections Median days of infectious-related clinical findings, warranting antibiotic initiation: 7	YES - 33 patients with confirmed infection - Antibiotics used - Median days of antibiotic duration: 17 - 17% of patients with confirmed infection also had malignancy the treatment of which was delayed due to the prolonged treatment of infection	NO - Not reported/described in the study	YES - 29 patients were investigated microbiologically - 8 patients had positive cultures
Steinfort D.P. et al. [42]	Prospective study Single-centered 43 patients Post-procedural blood sampling in 60 min for blood cultures Sample of the EBUS TBNA needle was also obtained Inflammatory biomarkers utilized: none	Identification of the incidence of bacteremia and infectious complications associated with EBUS-TBNA	Bacteremia incidence: 3 cases, 7% None of them experienced any significant complications No significant correlation of the bacteremia to the size of the sampled lesion or to the underlying pathology The bacteremia rate was comparable to that induced by conventional bronchoscopy	NO	NO - Not reported/described in the study	NO - The blood culture pathogens identified were related to the oropharyngeal flora - Culture of TBNA needle washings was negative in all three cases of bacteremia
Magnini et al. [43]	Prospective study Single-centered 697 patients Inflammatory biomarkers utilized: none	Evaluation of major complications and outcomes post-EBUS TBNA and EUS-B TBNA within 30 d - Severe complications: Bleeding Infection Respiratory Failure - Other complications - unplanned hospital encounter - 30-day mortality - adverse events by procedure type - factors associated with adverse events	Severe complications were identified in 17 (2.4%) cases. Late complications (8.47%) occurred Median time of presentation: 14 d. Infectious complications primarily in malignancy cases Patients with low density areas in their lesions had increased likelihood to develop infectious complications.	YES - Infectious complications, that led to unplanned hospital encounters: 2.5% of the cases. - Infectious complication led to prolonged (>2 weeks) use of antibiotics and delayed the treatment of neoplasmatic disease.	NO - Not reported/described in the study	YES - Blood or other sample cultures not specified but correlation of worst outcome was evident to those that required antibiotics, most likely because they were already identified as cases more prone to infection (diabetes, low attenuation lesions/necrosis, purulent secretions)
Souma T. et al. [44]	Retrospective study Single centered 1045 patients Criteria for infectious element: respiratory exacerbation for >24 h: fever >37 °C cough sputum, chest pain, dyspnea elevation of WBC or CRP compared with pre-bronchoscopy levels imaging findings with accompanying need of antibiotics Inflammatory biomarkers utilized: pre- and post-procedural WBC, CRP	Identification of infectious complications, after EBUS-TBNA sampling for peripheral lesions via a guide sheath, within 4 weeks	Infectious complications incidence: 47 (4.47%) cases Need of antibiotics Main risk factors identified: cavitation of lesion low-density areas in the lesion bronchial stenosis. Use of prophylactic antibiotics—before or after the procedure, in 102 patients—could not provide reliable results regarding the efficacy in preventing post-procedural infectious complications	YES - pneumonia (24) - intratumoral infection (14) - lung abscess (3) - pleuritis (3) - empyema (3)	YES Increased CRP, WBC	YES - Bronchial washings cultures: 47 positive 27 normal flora
Minami D. et al. [45]	Retrospective Single-centered 80 patients’ EBUS TBNA sampling Two groups of patients: 60 intubated 20 non-intubated Fever was assessed EBUS TBNA needle wash cultures processed Inflammatory biomarkers utilized: none	Identification of procedure-related infectious reactions and correlation with EBUS-TBNA needle wash cultures comparing the intubated and non-intubated groups	Positive EBUS-TBNA needle wash cultures: Intubated group: only 2 cases (3.3%) Non-intubated group: all 20 cases (100%). Fever occurred: Intubated group: 6 (10%) Non-intubated group: only 2 cases (10%). Fever development was equal in both groups despite the fact that contamination of EBUS-TBNA needle is less likely with the use of intubation	NO Fever was not associated with any clinical features	NO - Not reported/described in the study	NO - EBUS-TBNA needle wash cultures were positive in all cases of the non-intubated group and in two of the intubated group, but no significant correlation with infectious clinical reactions was identified
Mitja S. et al. [47]	Prospective Multi-centered 370 patients EBUS TBNA sampling Two groups of patients: 245 with risk factors 125 without risk factors (control group) 30-day follow up: days 2, 14, 21, 30 Fever (>38 °C) assessed initially, every 8 h, for 48 h Blood samples obtained 30 min post-procedure Inflammatory biomarkers utilized: WBC, ESR, CRP, procalcitonin Blood cultures and bronchial aspirate cultures processed	Identification of EBUS TBNA –related infectious complications and of potential risk factors	Infectious incidence: 15 cases (4.05%) 14 cases (5.71%) with risk factors 1 case (0.8%), from the control group Stronger risk factors: - Lesion’s necrosis - TBNA punctures >10	YES Fever presentation: - Nine patients (2.43%) presented fever immediately after EBUS-TBNA 6 of the 9 cases had self-limited fever 3 of the 9 cases developed infectious complication (pneumonia) - 14 patients (3.78%) developed fever during the 48 h after EBUS-TBNA self-limited 7 of the 14 cases had self-limited fever 7 of the 14 cases developed infectious complication related to infectious complication: mediastinitis (1) pneumonia (1) obstructive pneumonia (2) respiratory tract infection (2) All required antibiotics and infectious complication resolved without any more severe complication	NO - Not reported/described in the study (despite mention within the data collection)	YES - Blood culture positive in one case (0.27%) - Bronchial aspirate culture positive in 25 cases (6.75%)

**Table 3:** Useful clinical biomarkers for infection: advantages and disadvantages [70].

Biomarker	Specificity Bacterial Infection	Sensitivity Inflammation	Advantages	Disadvantages
WBC	low	high	Simple and non expensive	Sensitivity for bacteria Non-specific for bacterial infection All inflammation & infections Disease states
CRP	moderate	moderate	Non expensive Moderately specific	All inflammation and infections Slow induction (peak > 24 h) No correlation with severity
Lactate	low	low	Inexpensive Reliable marker of perfusion Prognosis > Sepsis	Must be in sepsis to be elevated Very poor specificity for bacterial infection
Fever	low	low	Inexpensive Readily available	No specificity to bacteria Affected by >1180 drugs and/or disease states
Procalcitonin	high	low	Specificity for bacteria Favorable kinetics Rise/half-life Correlates with severity of illness Antibiotic use	Education Instrument for Lab More expensive than WBC, CRP, and lactate
